# Implications of improved Higgs mass calculations for supersymmetric models

**DOI:** 10.1140/epjc/s10052-014-2809-3

**Published:** 2014-03-18

**Authors:** O. Buchmueller, M. J. Dolan, J. Ellis, T. Hahn, S. Heinemeyer, W. Hollik, J. Marrouche, K. A. Olive, H. Rzehak, K. J. de Vries, G. Weiglein

**Affiliations:** 1 High Energy Physics Group, Blackett Lab., Imperial College, Prince Consort Road, London, SW7 2AZ UK; 2 Theory Group, SLAC National Accelerator Lab., 2575 Sand Hill Road, Menlo Park, CA 94025-7090 USA; 3 Theoretical Particle Physics and Cosmology Group, Department of Physics, King’s College London, London, WC2R 2LS UK; 4 Theory Division, CERN, 1211 Geneva 23, Switzerland; 5 Max-Planck-Institut für Physik, Föhringer Ring 6, 80805 Munich, Germany; 6 Instituto de Física de Cantabria (CSIC-UC), 39005 Santander, Spain; 7 William I. Fine Theoretical Physics Institute, School of Physics and Astronomy, University of Minnesota, Minneapolis, MN 55455 USA; 8 Physikalisches Institut, Albert-Ludwigs-Universität Freiburg, 79104 Freiburg, Germany; 9 DESY, Notkestrasse 85, 22607 Hamburg, Germany

## Abstract

We discuss the allowed parameter spaces of supersymmetric scenarios in light of improved Higgs mass predictions provided by FeynHiggs 2.10.0. The Higgs mass predictions combine Feynman-diagrammatic results with a resummation of leading and subleading logarithmic corrections from the stop/top sector, which yield a significant improvement in the region of large stop masses. Scans in the pMSSM parameter space show that, for given values of the soft supersymmetry-breaking parameters, the new logarithmic contributions beyond the two-loop order implemented in FeynHiggs tend to give larger values of the light CP-even Higgs mass, $M_h$, in the region of large stop masses than previous predictions that were based on a fixed-order Feynman-diagrammatic result, though the differences are generally consistent with the previous estimates of theoretical uncertainties. We re-analyse the parameter spaces of the CMSSM, NUHM1 and NUHM2, taking into account also the constraints from CMS and LHCb measurements of $\mathrm{BR}(B_s \rightarrow \mu ^+\mu ^-)$and ATLAS searches for $/\!\!\!E_T$ events using 20/fb of LHC data at 8 TeV. Within the CMSSM, the Higgs mass constraint disfavours ${\tan \beta }\lesssim 10$, though not in the NUHM1 or NUHM2.

## Introduction

The ATLAS and CMS experiments did not discover supersymmetry (SUSY) during the first, low-energy LHC run at 7 and 8 TeV. However, an optimist may consider that the headline discovery of a Higgs boson weighing ${\sim }126 \,\hbox {GeV}$ [1, 2] has provided two additional pieces of indirect, circumstantial evidence for SUSY, beyond the many previous motivations. One piece of circumstantial evidence is provided by the Higgs mass, which falls within the range ${\lesssim }135 \,\hbox {GeV}$ calculated in the minimal SUSY extension of the Standard Model (MSSM) for masses of the SUSY particles around 1 TeV [3–15]. The other piece of circumstantial evidence is provided by measurements of Higgs couplings, which do not display any significant deviations from Standard Model (SM) predictions at the present level of experimental accuracy. This disfavours some composite models but is consistent with the predictions of simplified SUSY models such as the constrained MSSM (CMSSM) [16–25] with universal input soft SUSY-breaking masses $m_0$ for scalars, $m_{1/2}$ for fermions as well as $A_0$, the soft SUSY-breaking trilinear coupling and NUHM models that have non-universal soft SUSY-breaking contributions to Higgs supermultiplet masses: see [26–30] and [31] for a review.

That said, the absence of SUSY in the first LHC run and the fact that the Higgs mass is in the upper part of the MSSM range both suggest, within simple models such as the CMSSM and NUHM (see, e.g., [32, 33]) as well as in the pMSSM, that the SUSY particle mass scale may be larger than had been suggested prior to the LHC, on the basis of fine-tuning arguments and in order to explain the discrepancy between calculations of $(g-2)_\mu$ within the SM and the experimental measurement [34]. A relatively large SUSY particle mass scale also makes it easier to reconcile SUSY with the experimental measurement of $\mathrm{BR}(B_s \rightarrow \mu ^+\mu ^-)$ [35–37], particularly if ${\tan \beta }$ (the ratio of SUSY Higgs vacuum expectation values, v.e.v.s) is large.

The mathematical connection between the Higgs mass and the SUSY particle spectrum is provided by calculations of the lightest SUSY Higgs mass $M_h$ in terms of the SUSY particle spectrum [3–11, 14, 15]: see [38–40] for reviews. As is well known, one-loop radiative corrections allow $M_h$ to exceed $M_Z$ by an amount that is logarithmically sensitive to such input parameters as the top squark masses $m_{\tilde{t}}$ in the pMSSM, or the universal $m_{1/2}$ and $m_0$ masses in the CMSSM and NUHM. Inverting this calculation, the inferred values of $m_{\tilde{t}}$, or $m_{1/2}, m_0$ and $A_0$ are exponentially sensitive to the measured value of $M_h$. For this reason, it is essential to make available and use the most accurate calculations of $M_h$ within the MSSM, and to keep track of the unavoidable theoretical uncertainties in these calculations due to unknown higher-order corrections, which are now larger than the experimental measurement error.

Several codes to calculate $M_h$ are available [41–48]. In terms of low-energy parameters, the most advanced calculation is provided by FeynHiggs [14, 49–52]. The differences between the codes are in the few GeV range for relatively light SUSY spectra, but they may become larger for higher third family squark masses and values of $m_{1/2}, m_0$ and $A_0$. This is particularly evident in the phenomenological MSSM (pMSSM), where the soft supersymmetry-breaking inputs to the SUSY spectrum codes are specified at a low scale, close to the physical masses of the supersymmetric particles.

In this paper we revisit the constraints on the CMSSM and NUHM parameter spaces imposed by the Higgs mass measurement using the significantly improved 2.10.0 version of the FeynHiggs code [49–53] that has recently been released. We situate our discussion in the context of a comparison between this and the earlier version FeynHiggs 2.8.6, which has often been used in phenomenological studies of SUSY parameter spaces (e.g., in [54]), as well as with SOFTSUSY 3.3.9 [41]. We also discuss the implications for constraints on SUSY model parameters. Updating previous related analyses [32, 33], we also take into account the complementary constraint on the CMSSM and NUHM parameter spaces imposed by the recent experimental measurement of $\mathrm{BR}(B_s \rightarrow \mu ^+\mu ^-)$, and we incorporate the 95 % CL limit on $m_{1/2}$ and $m_0$ established within the CMSSM by ATLAS following searches for missing transverse energy, $/\!\!\!E_T$, events using 20/fb of LHC data at 8 TeV [55].

The layout of this paper is as follows. In Sect. 2 we first summarise the main improvements between the results implemented in FeynHiggs 2.8.6 and 2.10.0, and then we present some illustrative results in the pMSSM, discussing the numerical differences between calculations made using FeynHiggs versions 2.8.6 and 2.10.0. We then display in Sect. 3 some representative parameter planes in the CMSSM, NUHM1 and NUHM2, discussing the interplay between the different experimental constraints including $\mathrm{BR}(B_s \rightarrow \mu ^+\mu ^-)$as well as $M_h$. Section 4 contains a discussion of the variations between the predictions of $M_h$ made in global fits to CMSSM and NUHM1 model parameters using different versions of FeynHiggs and SOFTSUSY. Finally, Sect. 5 summarises our conclusions.

## Comparisons of Higgs mass calculations within the general MSSM

### The improved Higgs mass calculation in FeynHiggs 2.10.0

The evaluation of Higgs boson masses in the MSSM, in particular of the mass of the lightest Higgs boson, $M_h$, has recently been improved for larger values of the scalar top mass scale. This new evaluation has been implemented in the code FeynHiggs 2.10.0, whose details can be found in [53]. Here we just summarise some salient points.

The code FeynHiggs provides predictions for the masses, couplings and decay properties of the MSSM Higgs bosons at the highest currently available level of accuracy as well as approximations for LHC production cross sections (for MSSM Higgs decays see also [56] and references therein). The evaluation of Higgs boson masses within FeynHiggs is based on a Feynman-diagrammatic calculation of the Higgs boson self-energies. By finding the higher-order corrected poles of the propagator matrix, the loop-corrected Higgs boson masses are obtained.

The principal focus of the improvements in FeynHiggs 2.10.0 has been to attain greater accuracy for large stop masses. The versions of FeynHiggs as used, e.g., previously in [54] included the full one-loop and the leading and subleading two-loop corrections to the Higgs boson self-energies (and thus to $M_h$). The new version, FeynHiggs 2.10.0 [53], which is used for the evaluations here, contains in addition a resummation of the leading and next-to-leading logarithms of type $\log (m_{\tilde{t}}/m_{t})$ in all orders of perturbation theory, which yields reliable results for $m_{\tilde{t}}, M_A\gg M_Z$. To this end the two-loop Renormalisation-Group Equations (RGEs) [57, 58] have been solved, taking into account the one-loop threshold corrections to the quartic coupling at the SUSY scale: see [59] and references therein. In this way at $n$-loop order the terms

1 $$\begin{aligned} {\sim }\log ^n (m_{\tilde{t}}/m_{t}), \quad {\sim } \log ^{n-1}(m_{\tilde{t}}/m_{t}) \end{aligned}$$

are taken into account. The resummed logarithms, which are calculated in the $\overline{\mathrm{MS}}$ scheme for the scalar top sector, are matched to the one- and two-loop corrections, where the on-shell scheme had been used for the scalar top sector. The first main difference between FeynHiggs 2.10.0 and previous versions occurs at three-loop order. As we shall see, FeynHiggs 2.10.0 yields a larger estimate of $M_h$ for stop masses in the multi-TeV range and a correspondingly improved estimate of the theoretical uncertainty, as discussed in [53]. The improved estimate of the uncertainties arising from corrections beyond two-loop order in the top/stop sector is adjusted such that the impact of replacing the running top-quark mass by the pole mass (see [14]) is evaluated only for the non-logarithmic corrections rather than for the full two-loop contributions implemented in FeynHiggs.

Other codes such as SoftSusy [41], SPheno [42, 43] and SuSpect [44] implement a calculation of the Higgs masses based on a $\overline{\mathrm{DR}}$ renormalisation of the scalar quark sector^1^. These codes contain the full one-loop corrections to the MSSM Higgs masses and implement the most important two-loop corrections. In particular, SoftSusy contains the ${\mathcal O}(\alpha _t^2)$, ${\mathcal O}(\alpha _b\alpha _\tau )$, ${\mathcal O}(\alpha _b^2)$, ${\mathcal O}(\alpha _b\alpha _s)$, ${\mathcal O}(\alpha _t \alpha _s)$, ${\mathcal O}(\alpha _\tau ^2)$ and ${\mathcal O}(\alpha _t \alpha _b)$ corrections of [11–13, 15] evaluated at zero external momentum for the neutral Higgs masses. These codes do not contain the additional resummed higher-order terms included in FeynHiggs 2.10.0. We return in Sect. 4 to a comparison between SoftSusy3.3.9 and FeynHiggs2.10.0.

More recently a calculation of $M_h$ taking into account leading three-loop corrections of $\mathcal{O}(\alpha _t\alpha _s^2)$ has became available, based on a $\overline{\mathrm {DR}}$ or a “hybrid” renormalisation scheme for the scalar top sector, where the numerical evaluation depends on the various SUSY mass hierarchies, resulting in the code H3m [46–48], which adds the three-loop corrections to the FeynHiggs result. A brief comparison between FeynHiggs and H3m can be found in [53, 60].

A numerical analysis in the CMSSM including leading three-loop corrections to $M_h$ (with the code H3m) was presented in [60]. It was shown that the leading three-loop terms can have a strong impact on the interpretation of the measured Higgs mass value in the CMSSM. Here, with the new version of FeynHiggs, we go beyond this analysis by including (formally) subleading three-loop corrections as well as a resummation to all orders of the leading and next-to-leading logarithmic contributions to $M_h$; see above.

### Comparing the improved Higgs mass calculation in FeynHiggs 2.10.0 with FeynHiggs 2.8.6

In the following we examine the effect of including the resummation of leading and subleading logarithmic corrections from the (scalar) top sector in the pMSSM. We compare the new FeynHiggs version 2.10.0 with a previous one, 2.8.6, where the only relevant difference in the Higgs mass calculation between the two codes consists of the aforementioned resummation effects. (A comparison including SOFTSUSY can be found in Sect. 4.) These corrections are most sensitive to the soft SUSY-breaking parameters in the stop sector, $m_{\widetilde{q_3}}$ in the diagonal entry (which we assume here to be equal for left- and right-handed stops) and the trilinear coupling $A_t$. To have direct control over these two parameters, we consider a 10-parameter incarnation of the MSSM, denoted as the pMSSM10. In the pMSSM10 we set the squark masses of the first two generations to a common value $m_{\widetilde{q}_{12}}$, the third-generation squark mass parameters to a different value $m_{\widetilde{q}_3}$, the slepton masses to $m_{\widetilde{l}}$ and the trilinear couplings $A_t= A_b = A_{\tau } = A$. The remaining parameters of the pMSSM10 are the soft SUSY-breaking parameters in the gaugino sectors, $M_1, M_2, M_3$, the Higgs mixing parameter $\mu$, the CP-odd Higgs mass scale $M_A$ as well as ${\tan \beta }$.

We generate 1000 random sets of the eight parameters $m_{\widetilde{q}_{12}}m_{\widetilde{l}}, M_1, M_2, M_3, {\tan \beta }, \mu$ and $M_A$, without regard to the experimental constraints. For each of these sets we vary $m_{\widetilde{q}_3}=0.5, 1, 2, 3, 4$ and 5 TeV and $A/m_{\widetilde{q}_3}=0, \pm 1.0, \pm 2.0, \pm 2.4$, and we calculate the corresponding spectra using SOFTSUSY-3.3.9. Using these spectra, we calculate $M_h$ with FeynHiggs 2.8.6 and FeynHiggs 2.10.0. We stress that the pMSSM10 spectra are only meant to illustrate the size of the corrections as a function of $m_{\widetilde{q_3}}$ and the trilinear coupling $A$, and we do not necessarily correspond to phenomenologically interesting regions of parameter space.

**Fig. 1:**
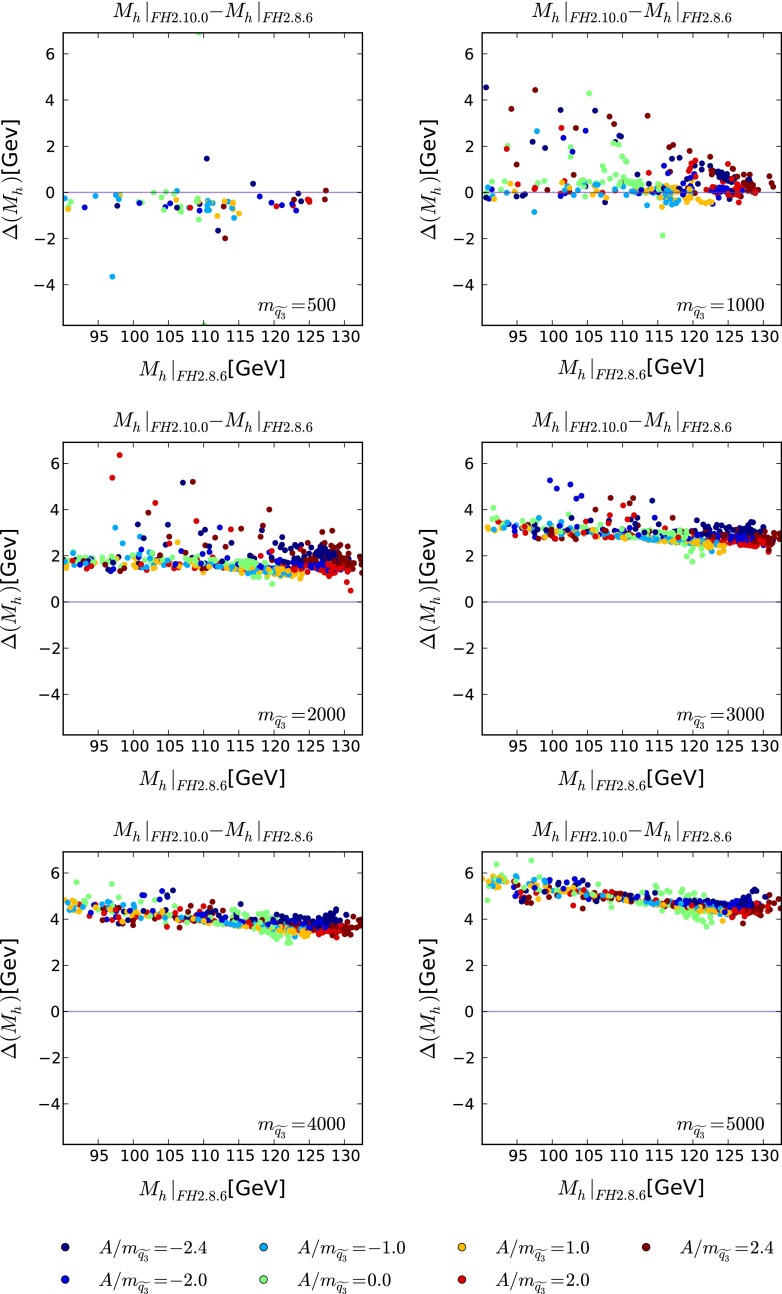
The differences between $M_h$ calculated using FeynHiggs 2.10.0 and FeynHiggs 2.8.6, as a function of the FeynHiggs 2.8.6 value, for third-generation squark masses $m_{\widetilde{q}_3}= 0.5$ TeV (*upper left*), 1 TeV (*upper right*), 2 TeV (*middle left*), 3 TeV (*middle right*), 4 TeV (*lower left*) and 5 TeV (*lower right*)

The sizes of the corrections from the (scalar) top sector are given by the differences $(M_h|_{\mathrm{FH}2.10.0}-M_h|_{\mathrm{FH}2.8.6})$ shown in Fig. 1 as functions of $M_h|_{\mathrm{FH}2.8.6}$. The different panels in this figure correspond to the different third-generation squark masses $m_{\widetilde{q}_3}=0.5$ TeV (upper left), 1 TeV (upper right), 2 TeV (middle left), 3 TeV (middle right), 4 TeV (lower left) and 5 TeV (lower right), whereas the colours dark blue, blue, light blue, light green, orange, red and dark red correspond to $A/m_{\widetilde{q}_3}=-2.4, -2.0, -1.0, 0.0, 1.0, 2.0, 2.4$, respectively. At low stop masses of around 500 GeV we see that the resummation corrections are $\mathcal{O}(0.5)$ GeV, whereas with increasing stop masses they may become as large as 5 GeV. The dependence on $A/m_{\widetilde{q}_3}$ is less significant. We also note that, for similar values of $m_{\widetilde{q}_3}$, the resummation corrections tend to be smaller for models yielding $M_h\sim 125 \,\hbox {GeV}$ than for models yielding smaller values of $M_h$.

The latter effect is related to the (random) choice of $M_A$ and ${\tan \beta }$, with lower $M_h$ values corresponding to lower $M_A$ and smaller ${\tan \beta }$. If the $M_h$ value without resummed corrections, i.e., from FeynHiggs 2.8.6, is smaller, the newly added correction, which is independent of $M_A$ and ${\tan \beta }$ has a larger effect. We should furthermore mention that the size of the resummed correction stays (mostly) within the previously predicted estimate for the theoretical uncertainties due to missing higher-order corrections. Consequently, a point in the MSSM parameter space that has a Higgs mass value of, for instance, $125 \,\hbox {GeV}$ as evaluated by FeynHiggs 2.10.0, should not have been excluded on the basis of a lower $M_h$ as evaluated using FeynHiggs 2.8.6. However, the parallel reduction in the theory uncertainty in FeynHiggs 2.10.0 leads to a more precise restriction on the allowed MSSM parameter space.

## Examples of CMSSM and NUHM parameter planes

In our exploration of the FeynHiggs 2.10.0 results for $M_h$, we discuss their interplay with other experimental constraints, notably $\mathrm{BR}(B_s \rightarrow \mu ^+\mu ^-)$ and the ATLAS search for $/\!\!\!E_T$ events with 20/fb of data at 8 TeV. In this section, results were produced using SSARD [61] coupled to FeynHiggs. These results update those in [32] for the CMSSM and [33] for the NUHM. In the case of the CMSSM, we consider several $(m_{1/2}, m_0)$ planes for fixed values of ${\tan \beta }$ and $A_0/m_0$, all with $\mu > 0$. In the NUHM1 model we also display two $(m_{1/2}, m_0)$ planes for fixed values of ${\tan \beta }$ and $A_0/m_0$, one with fixed $\mu = 500 \,\hbox {GeV}$ and one with fixed $M_A= 1000 \,\hbox {GeV}$, and two $(\mu , m_0)$ planes with fixed ${\tan \beta }, m_{1/2}$ and $A_0/m_0$. In the NUHM2 we display two $(\mu , M_A)$ planes with fixed ${\tan \beta }, m_{1/2}, m_0$ and $A_0/m_0$. We also present one example of a $(m_{1/2}, m_0)$ plane in the minimal supergravity (mSUGRA) model, in which the electroweak vacuum conditions fix ${\tan \beta }$ as a function of $m_{1/2}, m_0$ and $A_0$.

We adopt the following conventions in all these figures. Regions where the LSP is charged are shaded brown, those where there is no consistent electroweak vacuum are shaded mauve, regions excluded by $\mathrm{BR}(b \rightarrow s \gamma )$ measurements at the 2-$\sigma$ level are shaded green,^2^ those favoured by the SUSY interpretation of $(g-2)_\mu$ are shaded pink, with lines indicating the $\pm 1\sigma$ (dashed) and $\pm 2\sigma$ ranges (solid),^3^ and strips with an LSP density appropriate to make up all the cold dark matter are shaded dark blue. For reasons of visibility, we shade strips where $0.06 < \Omega _\chi h^2 < 0.2$, but when we quote ranges of consistency we require that the relic density satisfies the more restrictive relic density bound $0.115 < \Omega _\chi h^2 < 0.125$ [70]. The 95 % CL limit from the ATLAS $/\!\!\!E_T$ search is shown as a continuous purple contour,^4^ and the 68 and 95 % CL limits from the CMS and LHCb measurements of $\mathrm{BR}(B_s \rightarrow \mu ^+\mu ^-)$ are shown as continuous green contours. Finally, the labelled continuous black lines are contours of $M_h$ calculated with FeynHiggs 2.10.0, and the dash-dotted red lines are contours of $M_h$ calculated with FeynHiggs 2.8.6 (as used, e.g., in [32, 33, 54]), which we use for comparison.

### The CMSSM

**Fig. 2:**
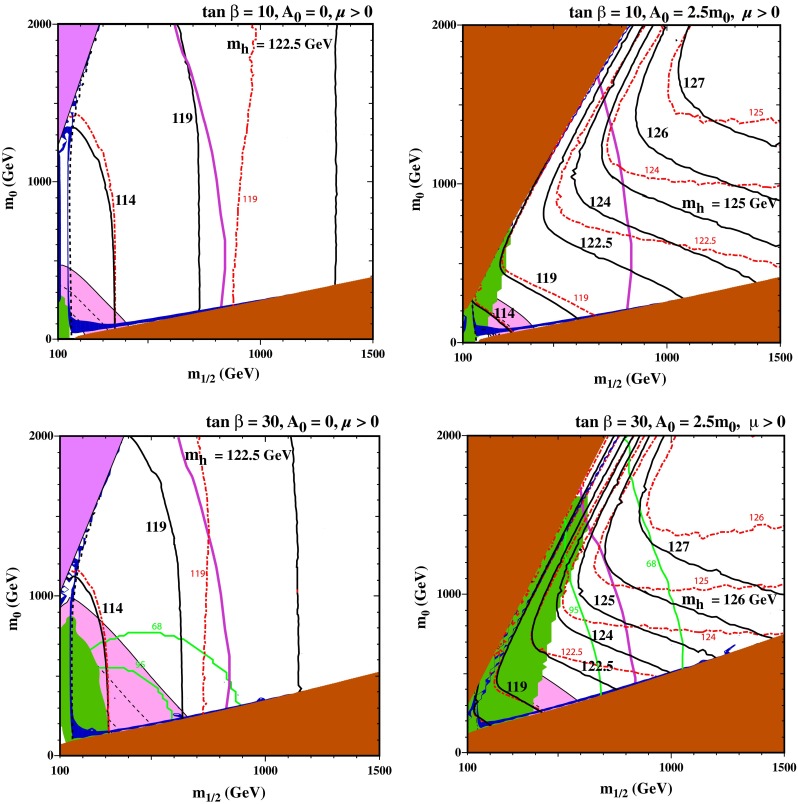
The allowed regions in the $(m_{1/2}, m_0)$ planes for ${\tan \beta }= 10$ and $A_0 = 0$ (*upper left*), ${\tan \beta }= 10$ and $A_0 = 2.5 m_0$ (*upper right*), ${\tan \beta }= 30$ and $A_0 = 0$ (*lower left*) and ${\tan \beta }= 30$ and $A_0 = 2.5 m_0$ (*lower right*). The line styles and shadings are described in the text. The section of the *dark blue* coannihilation strip in the *lower right panel* in the range $m_{1/2} \in (840, 1050) \,\hbox {GeV}$ is compatible with the constraints from $\mathrm{BR}(B_s \rightarrow \mu ^+\mu ^-)$ (*green line*) and the ATLAS 20/fb $/\!\!\!E_T$ search (*purple line*), as well as with the LHC $M_h$ measurement. Better consistency with all the constraints (except $(g-2)_\mu$) is found if the improved FeynHiggs 2.10.0 code is used, for ${\tan \beta }= 30$ and $A_0 = 2.5 m_0$

Figure 2 displays four examples of $(m_{1/2}, m_0)$ planes for relatively low values of ${\tan \beta }$. We see in the upper left panel for ${\tan \beta }= 10$ and $A_0 = 0$ that the contour for $M_h= 114 \,\hbox {GeV}$ (the lower limit set by the LEP experiments) changes very little between FeynHiggs 2.8.6 and 2.10.0, whereas that for 119 GeV is shifted by $\Delta m_{1/2} \sim - 150 \,\hbox {GeV}$ in the region of the stau-coannihilation strip at low $m_0$. The ATLAS 20/fb $/\!\!\!E_T$ limit on $m_{1/2}$ excludes robustly a SUSY solution to the $(g-2)_\mu$ discrepancy in this particular CMSSM scenario, but neither $b \rightarrow s \gamma$ nor $B_s \rightarrow \mu ^+ \mu ^-$ has any impact on the allowed section of the dark matter strip, which extends to $m_{1/2} \sim 900 \,\hbox {GeV}$ in this case. However, none of it is compatible with the measured value of $M_h$, even with the higher value and the correspondingly smaller theory uncertainty as evaluated by FeynHiggs 2.10.0 which is about $\pm 0.8 \,\hbox {GeV}$ near the endpoint of the strip. There is a mauve region at small $m_{1/2}$ and large $m_0$ where the electroweak vacuum conditions cannot be satisfied, adjacent to which there is a portion of a focus-point strip, excluded by the ATLAS $/\!\!\!E_T$ search, where $M_h$ is smaller than the measured value.

In the upper right panel of Fig. 2, which displays the case ${\tan \beta }= 10$ and $A_0 = 2.5 m_0$, we see that the FeynHiggs 2.10.0 $M_h= 119 \,\hbox {GeV}$ contour intersects the stau-coannihilation strip when $m_{1/2} \sim 600 \,\hbox {GeV}$ (a shift of less than 100 GeV in $m_{1/2}$ compared to FeynHiggs 2.8.6) and the tip of the strip corresponds to $M_h\sim 122 \,\hbox {GeV}$. The experimental value of $M_h$ lies somewhat outside the range around this value that is allowed by the uncertainty estimated in FeynHiggs 2.10.0, which is about $1.0 \,\hbox {GeV}$ at this point. Consequently, although the use of FeynHiggs 2.10.0 reduces significantly the tension with the measurement of $M_h$ for this value of ${\tan \beta }$ in the CMSSM, it seems that this model requires a larger value of ${\tan \beta }$.

We note in this case the appearance of a brown region in the upper left part of the plane, where the lighter scalar top is the LSP (or tachyonic), with an adjacent stop-coannihilation strip. We find $M_h< 122 \,\hbox {GeV}$ in the displayed section of the strip where $m_0 < 2000 \,\hbox {GeV}$, but larger values of $M_h$ can be found at larger $m_0$, which may be compatible with the LHC measurement, within the uncertainties. For example, at $m_{1/2} = 1500$ GeV, the stop-coannihilation strip is found at $m_0 \simeq 3450 \,\hbox {GeV}$ and the Higgs mass there computed with FeynHiggs 2.10.0 is $M_h\simeq 125 \,\hbox {GeV}$, substantially higher than the value of 121 GeV found in FeynHiggs 2.8.6, though with a larger uncertainty of 2 GeV.

The lower left panel of Fig. 2 displays the $(m_{1/2}, m_0)$ plane for ${\tan \beta }= 30$ and $A_0 = 0$. Compared with the ${\tan \beta }= 10, A_0 = 0$ case, the Higgs mass contours are similar, though shifted somewhat to lower $m_{1/2}$. The focus-point region is found at slightly larger $m_{1/2}$ but is not very different from the ${\tan \beta }= 10$ case. We note also the appearance of the (green) 68 and 95 % CL constraints from $\mathrm{BR}(B_s \rightarrow \mu ^+\mu ^-)$, though the constraints from the ATLAS $/\!\!\!E_T$ search and (particularly) $M_h$ are more important. Although the stau-coannihilation strip extends to slightly higher values of $m_{1/2} \sim 1000 \,\hbox {GeV}$ when $A_0 = 0$, the Higgs mass at the endpoint is still only $122 \pm 0.8 \,\hbox {GeV}$. It is well known that the calculated value of $M_h$ increases with the value of $A_0$, and compatibility with the LHC measurement for this value of ${\tan \beta }$ requires a larger value of $A_0$.

Accordingly, in the lower right panel of Fig. 2 we show the case of ${\tan \beta }= 30$ and $A_0 = 2.5 m_0$. As expected, the situation along the stau-coannihilation strip is much more favourable for $M_h$. At the end point of the stau-coannihilation strip, which is now at about $m_{1/2} \simeq 1250 \,\hbox {GeV}$, according to the improved FeynHiggs 2.10.0 calculation the Higgs mass is $M_h\simeq 125.2 \pm 1.1 \,\hbox {GeV}$, quite consistent with LHC measurement, whereas the previous version of FeynHiggs would have yielded $M_h\approx 123.4\, \pm \, 2.7 \,\hbox {GeV}$. This point is also compatible with the 68 % CL limit from $\mathrm{BR}(B_s \rightarrow \mu ^+\mu ^-)$. The 95 % CL upper limit on $\mathrm{BR}(B_s \rightarrow \mu ^+\mu ^-)$ requires $m_{1/2} \gtrsim 700 \,\hbox {GeV}$, already placing a SUSY interpretation of $(g-2)_\mu$ “beyond reach”, and the ATLAS 20/fb $/\!\!\!E_T$ search requires $m_{1/2} > 840 \,\hbox {GeV}$.

In the upper left corner of the plane, we again see a stop LSP region with a stop-coannihilation strip of acceptable relic density due running along its side. As in the case of the ${\tan \beta }= 10$, the strip as shown here corresponds to values of $M_h$ that are too low. However, at larger $m_0$, this too would be acceptable. At $m_{1/2} = 1500 \,\hbox {GeV}$ and $m_0 = 3750 \,\hbox {GeV}$, for example, we find $M_h\simeq 124 \pm 2 \,\hbox {GeV}$ with FeynHiggs 2.10.0, whereas FeynHiggs 2.8.6 would have yielded $M_h\lesssim 120 \,\hbox {GeV}$ albeit with an uncertainty of $\pm 5 \,\hbox {GeV}$. Thus, in the CMSSM with ${\tan \beta }= 30$ and $A_0 = 2.5 m_0$ there are two regions of compatibility with the LHC measurement of $M_h$ once the improved FeynHiggs 2.10.0 calculation of $M_h$ is taken into account.

**Fig. 3:**
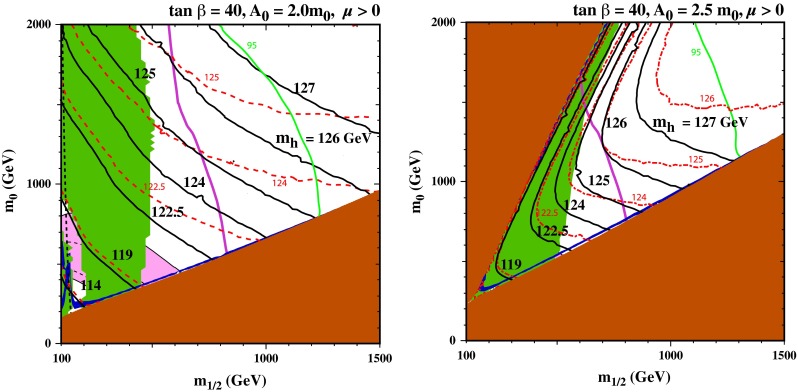
The allowed regions in the $(m_{1/2}, m_0)$ planes for ${\tan \beta }= 40$ and $A_0 = 2 m_0$ (*left*), ${\tan \beta }= 40$ and $A_0 = 2.5 m_0$ (*right*). The line styles and shadings are described in the text. When ${\tan \beta }= 40$, consistency is found only if the improved FeynHiggs 2.10.0 code is used, for the $A_0 = 2 m_0$ case

Figure 3 displays some analogous $(m_{1/2}, m_0)$ planes for ${\tan \beta }= 40$. For $A_0 = 0$ (not shown), the plane would be qualitatively similar to that with ${\tan \beta }= 30$, though the constraint from $\mathrm{BR}(B_s \rightarrow \mu ^+\mu ^-)$ would be much stronger. In this case, the 95 % CL constraint would intersect the coannihilation strip at roughly $m_{1/2} = 950 \,\hbox {GeV}$. Instead, we show results for both $A_0 = 2 m_0$ and $2.5 m_0$. In the case $A_0 = 2 m_0$ (left), we see that the $\mathrm{BR}(B_s \rightarrow \mu ^+\mu ^-)$ 95 % CL constraint allows only a small section of the stau-coannihilation strip with $m_{1/2} \sim 1200 \,\hbox {GeV}$. (The 68 % limit is at significantly higher values of $m_{1/2}$, well past the endpoint of the coannihilation strip). In this case, the $\mathrm{BR}(B_s \rightarrow \mu ^+\mu ^-)$ constraint is significantly stronger than the LHC $/\!\!\!E_T$ constraint, and much of the region with $m_{1/2} < 500 \,\hbox {GeV}$ is also excluded by $b \rightarrow s \gamma$. Whereas the previous version of FeynHiggs would have yielded $M_h< 123.3 \pm 2.6 \,\hbox {GeV}$ near the tip of the stau-coannihilation strip, the improved FeynHiggs 2.10.0 calculation yields $M_h\sim 125.0 \pm 1.1 \,\hbox {GeV}$ in this region, so it may now also be considered compatible with all the constraints (except $(g-2)_\mu$).

In the right panel of Fig. 3, we show the case of ${\tan \beta }= 40$ and $A_0 = 2.5 m_0$. In this case, the $\mathrm{BR}(B_s \rightarrow \mu ^+\mu ^-)$ constraint also is only compatible with the endpoint of the stau-coannihilation strip, which is now at $m_{1/2} \sim 1250 \,\hbox {GeV}$, where the Higgs mass computed with FeynHiggs 2.10.0 is as large as 127 GeV.^5^ (Once again, the LHC $/\!\!\!E_T$ constraint on $m_{1/2}$ is weaker, as is the $b \rightarrow s \gamma$ constraint.) In the upper left corner at $m_0 \gg m_{1/2}$, we again see a stop LSP region and a stop-coannihilation strip running along its side. The part of the strip shown is excluded by $b \rightarrow s \gamma$, but compatibility is found at larger $m_0$. For $m_{1/2} = 1500 \,\hbox {GeV}$ and $m_0 = 4050 \,\hbox {GeV}$, the stop-coannihilation strip is compatible with both constraints on $B$ decays, but FeynHiggs 2.10.0 yields $M_h= 120 \,\hbox {GeV}$, albeit with a larger uncertainty ${\sim }2 \,\hbox {GeV}$.

We have also considered the larger value ${\tan \beta }= 55$, but we find in this case that the $\mathrm{BR}(B_s \rightarrow \mu ^+\mu ^-)$ constraint is incompatible with the dark matter constraint.

### The NUHM1

In the NUHM1, universality of the input soft SUSY-breaking gaugino, squark and slepton masses is retained, and the corresponding contributions to the Higgs multiplets are allowed to be different but assumed to be equal to each other. In this case, there is an additional free parameter compared with the CMSSM, which allows one to choose either the Higgs superpotential mixing parameter $\mu$ or the pseudoscalar mass $M_A$ as a free parameter while satisfying the electroweak vacuum conditions. Here and in the following we neglect the $(g-2)_\mu$ constraint, which is compatible with the ATLAS $/\!\!\!E_T$ searches only at around the $\pm 2.5{-}3\sigma$ level in the cases studied.

**Fig. 4:**
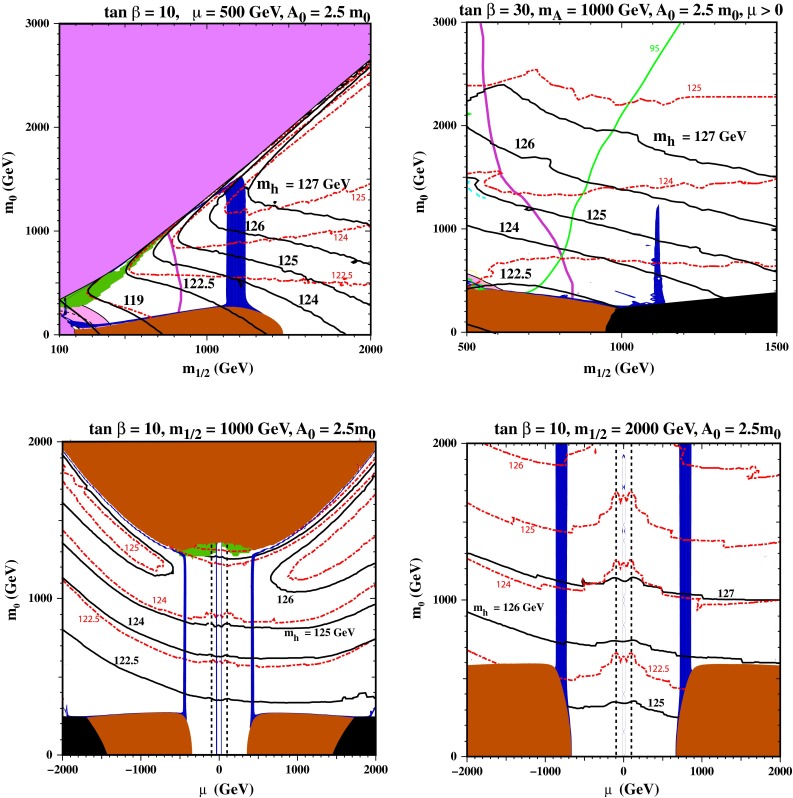
Examples of parameter planes in the NUHM1. Two $(m_{1/2}, m_0)$ planes shown in the upper panels have $A_0 = 2.5 m_0$ for $\tan \beta = 10$ and $\mu = 500 \,\hbox {GeV}$ (*left*) and $\tan \beta = 30$ and $M_A= 1000 \,\hbox {GeV}$ (*right*). Also shown are $(\mu , m_0)$ planes with ${\tan \beta }= 10$ and $m_{1/2} = 1000 \,\hbox {GeV}$ (*lower left*) and $m_{1/2} = 2000 \,\hbox {GeV}$ (*lower right*). In all the panels there are regions of consistency with all the experimental constraints if the improved FeynHiggs 2.10.0 code is used

The upper left panel of Fig. 4 displays the NUHM1 $(m_{1/2}, m_0)$ plane for ${\tan \beta }= 10, A_0 = 2.5 m_0$ and $\mu = 500 \,\hbox {GeV}$. In this case, we see that the stau-coannihilation strip at low $m_0$ is connected to the focus-point strip by a broader (dark blue) band with $m_{1/2} \sim 1200 \,\hbox {GeV}$ that is compatible with the astrophysical dark matter constraint. In this band, the composition of the LSP has a substantial Higgsino admixture that brings the relic density down into the astrophysical range, and its location depends on the assumed value of $\mu$. The value chosen here, $\mu = 500 \,\hbox {GeV}$, places this band beyond the ATLAS 20/fb $/\!\!\!E_T$ limit, and the $\mathrm{BR}(B_s \rightarrow \mu ^+\mu ^-)$ constraint is not important for this value of ${\tan \beta }$. Furthermore, we see from the $M_h$ contours that all this band is compatible with the Higgs mass measurement if the improved code FeynHiggs 2.10.0 is used. Only the upper part of this strip would have appeared consistent if the previous version of FeynHiggs had been used. This example shows that the freedom to vary $\mu$ within the NUHM1 opens up many possibilities to satisfy the experimental constraints, e.g., a lower value of ${\tan \beta }$ than was possible in the CMSSM.

The upper right panel of Fig. 4 displays the $(m_{1/2}, m_0)$ plane for ${\tan \beta }= 30, A_0 = 2.5 m_0$ and fixed $M_A= 1000 \,\hbox {GeV}$.^6^ In this case there is a spike at $m_{1/2} \sim 1100 \,\hbox {GeV}$ in which the dark matter density is brought down into the range allowed by astrophysics and cosmology by rapid LSP annihilations into the heavy Higgs bosons $H/A$, a mechanism that operates whenever $m_{\tilde{\chi }^0_{1}} \sim M_A/2$, namely ${\sim }500 \,\hbox {GeV}$ in this case. All of the spike is comfortably consistent with the ATLAS 20/fb $/\!\!\!E_T$ constraint and the upper limit on $\mathrm{BR}(B_s \rightarrow \mu ^+\mu ^-)$. We see that in the upper part of this spike FeynHiggs 2.10.0 yields a nominal value of $M_h\in (125, 126) \,\hbox {GeV}$, an increase of about 1.5 GeV over FeynHiggs 2.8.6, but lower parts of the spike may also be consistent with the LHC Higgs mass measurement, given the theoretical uncertainties. On the other hand, only limited consistency in the lower part of the strip would have been found with the previous version of FeynHiggs. This example shows that the freedom to vary $M_A$ within the NUHM1 opens up many possibilities to satisfy the experimental constraints.

In the lower left panel of Fig. 4 we display a different type of slice through the NUHM1 parameter space, namely a $(\mu , m_0)$ plane for fixed ${\tan \beta }= 10, m_{1/2} = 1000 \,\hbox {GeV}$ and $A_0 = 2.5 m_0$. With this choice of $m_{1/2}$, the ATLAS 20/fb $/\!\!\!E_T$ constraint is automatically satisfied throughout the plane, and with this choice of ${\tan \beta }$ the $\mathrm{BR}(B_s \rightarrow \mu ^+\mu ^-)$ constraint is also satisfied everywhere. We see two near-vertical dark blue bands where the relic LSP density falls within the cosmological range, again because of a large admixture of Higgsino in the LSP composition associated with the near-degeneracy of two neutralino mass eigenstates. These bands stretch between a stop LSP region at large $m_0$ and a stau LSP region at low $m_0$, which is flanked by charged slepton LSP regions at large $|\mu |$. We see that over much of this plane the value of $M_h$ calculated with FeynHiggs 2.10.0 is ${\sim }1 \,\hbox {GeV}$ higher than the 2.8.6 value. The upper parts of the dark blue bands again yield a nominal value of $M_h\in (125, 126) \,\hbox {GeV}$, and much of the rest of the bands may be compatible within the theoretical uncertainties.

The same is true in the lower right panel of Fig. 4, where we display an analogous $(\mu , m_0)$ plane for ${\tan \beta }= 10, m_{1/2} = 2000 \,\hbox {GeV}$ and $A_0 = 2.5 m_0$. Here we see that the stau LSP regions have expanded to larger $m_0$, and there are again near-vertical dark matter bands rising from them, whilst the stop LSP region has receded to larger $m_0$. In general, values of $M_h$ are larger than previously, with FeynHiggs 2.10.0 yielding nominal values ${\gtrsim }127 \,\hbox {GeV}$ for $m_0 > 1000 \,\hbox {GeV}$. This is roughly 3 GeV higher than found in FeynHiggs 2.8.6. In this case, values of $M_h$ as low as 125 GeV are attained only at the lower tips of the dark matter bands, very close to the stau LSP region with $m_0 \sim 300 \,\hbox {GeV}$. However, the entire bands are probably compatible with the LHC measurement of $M_h$ when the theoretical uncertainties are taken into account.

We conclude from the analysis in this section that values of $M_h\sim 125$ to 126 GeV are unexceptional in the NUHM1 and possible, e.g., for smaller values of ${\tan \beta }$ than in the CMSSM, though disfavouring a supersymmetric interpretation of $(g-2)_\mu$.

### The NUHM2

**Fig. 5:**
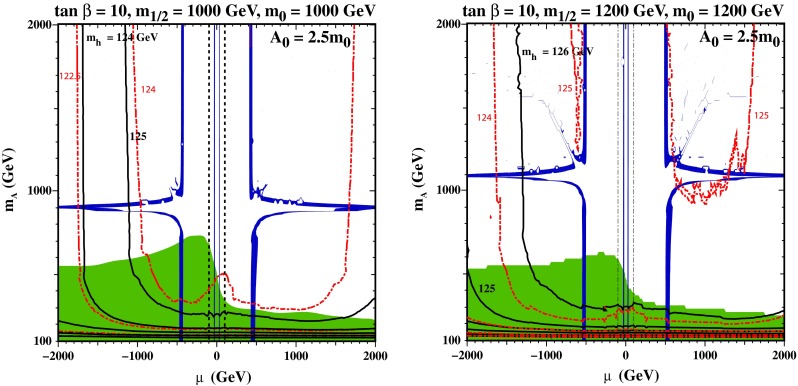
Examples of $(\mu , M_A)$ planes in the NUHM2 for $\tan \beta = 10$ and $A_0 = 2.5 m_0$, with $m_{1/2} = m_0 = 1000 \,\hbox {GeV}$ (*left*) and with $m_{1/2} = m_0 = 2000 \,\hbox {GeV}$ (*right*). Using the improved FeynHiggs 2.10.0 code, consistency with the measured value of $M_h$ is found over all the dark matter bands in both panels

In the NUHM2, the soft SUSY-breaking contributions to the masses of the two Higgs multiplets are allowed to vary independently, so there are two additional parameters compared to the CMSSM, which may be taken as $\mu$ and $M_A$. Figure 5 displays illustrative $(\mu , M_A)$ planes for fixed values of the other parameters ${\tan \beta }= 10, A_0 = 2.5 m_0$ and $m_{1/2} = m_0 = 1000 \,\hbox {GeV}$ (left), $m_{1/2} = m_0 = 1200 \,\hbox {GeV}$ (right). We see immediately that the $b \rightarrow s \gamma$ constraint is stronger for $\mu <0$ (which is one of the reasons that more studies have been made of models with $\mu >0$) and that $M_h$ is generally larger for $\mu > 0$ than for $\mu < 0$, if equal values of the other model parameters are chosen. The vertical dark matter strips correspond to large Higgsino admixtures, as in the NUHM1 examples discussed earlier, and the horizontal funnels are due to enhancement of LSP annihilation by direct-channel $H/A$ poles: these move to higher (lower) $M_A$ for larger (smaller) $m_{1/2}$, as seen by comparing the left and right panels of Fig. 5.

All the dark matter-compatible points in the left panel would correspond to values of $M_h$ consistent with the experimental measurements within the theoretical uncertainties. In this case, the shift in $M_h$ from FeynHiggs 2.8.6 to FeynHiggs 2.10.0 is about 1 GeV at $m_{1/2} = m_0 = 1000 \,\hbox {GeV}$ and somewhat larger at higher $m_{1/2}, m_0$ as seen in the right panel. In the right panel we see that typical nominal FeynHiggs 2.10.0 values of $M_h$ are larger than the measured value, though they are consistent with experiment, given the theoretical uncertainties.

### mSUGRA

Finally, we consider a scenario that is more restrictive than the CMSSM, namely minimal supergravity (mSUGRA). In this case, there is a universal input scalar mass $m_0$ equal to the gravitino mass $m_{3/2}$ and the soft bilinear and trilinear soft SUSY-breaking masses are related by $A_0 = (B_0 + 1) m_0$; see [31] for a review. The first constraint means that we do not have the luxury of assuming $m_{3/2}$ to be arbitrarily large, and there are regions of the $(m_{1/2}, m_0)$ plane where the LSP is necessarily the gravitino. The relation between $A_0$ and $B_0$ implies that ${\tan \beta }$ is determined at any point in the $(m_{1/2}, m_0)$ plane once $A_0$ is fixed.

**Fig. 6:**
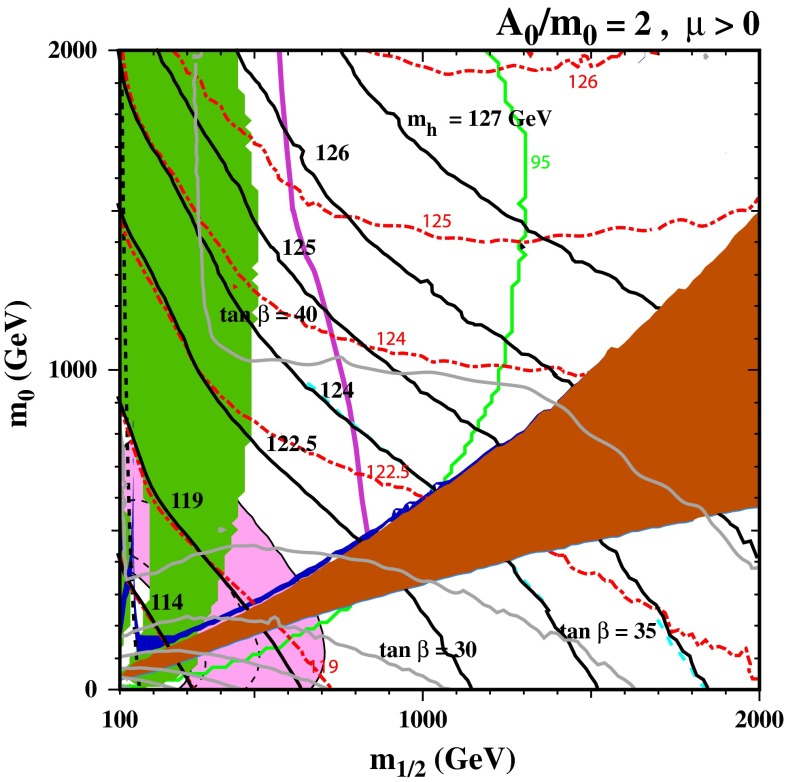
The allowed regions in the $(m_{1/2}, m_0)$ plane in a mSUGRA model with $A_0/m_0 = 2$. In addition to the line and shade descriptions found in the text, shown here are labelled *solid grey contours* showing the derived value of ${\tan \beta }$. Using the improved FeynHiggs 2.10.0 code, consistency with the measured value of $M_h$ is found near the tip of the stau-coannihilation strip

Both these features are visible in Fig. 6, where the $(m_{1/2}, m_0 = m_{3/2})$ plane for $A_0 = 2 m_0$ and $\mu > 0$ exhibits (grey) contours of ${\tan \beta }$ and a wedge where the LSP is the lighter stau, flanked by a neutralino LSP region at larger $m_0 = m_{3/2}$ and a gravitino LSP region at smaller $m_0 = m_{3/2}$. The ATLAS 20/fb $/\!\!\!E_T$ search is directly applicable only in the neutralino LSP region, and it requires reconsideration in the gravitino LSP region. In addition, in this region there are important astrophysical and cosmological limits on long-lived charged particles (in this case staus) that we do not consider here, so we concentrate on the neutralino LSP region above the stau LSP wedge. The ATLAS 20/fb $/\!\!\!E_T$ constraint intersects the dark matter coannihilation strip just above this wedge where $m_{1/2} \sim 850 \,\hbox {GeV}$, and the $\mathrm{BR}(B_s \rightarrow \mu ^+\mu ^-)$ constraint intersects the coannihilation strip at $m_{1/2} \sim 1050 \,\hbox {GeV}$, whereas the tip of the strip is at $m_{1/2} \sim 1250 \,\hbox {GeV}$. In this section of the coannihilation strip the nominal value of $M_h$ provided by the improved FeynHiggs 2.10.0 calculation is $\in (124, 125) \,\hbox {GeV}$, compatible with the experimental measurement within the theoretical uncertainties due to the 1–2 GeV shift in $M_h$ found in this new version of FeynHiggs.

## Higgs mass Predictions from global fits within the CMSSM and NUHM1

We saw in previous sections that different calculations of $M_h$ may differ significantly, particularly at large values of $m_{1/2}$ and/or $m_0$. With the improved $M_h$ calculation in FeynHiggs 2.10.0, the theory uncertainty has now been reduced to allow more precise $M_h$ evaluations also for larger values of the relevant SUSY parameters. Taking this into account, we found regions in the CMSSM that were compatible with the LHC measurement of $M_h$ and other constraints when the improved FeynHiggs 2.10.0 code is used, as well as broader possibilities for compatibility in the NUHM1 and NUHM2. In this Section we consider the possible implications for global fits to SUSY model parameters that include $M_h$ in the construction of the global likelihood function, concentrating for definiteness on the CMSSM and NUHM1 fits presented in [54].

**Fig. 7:**
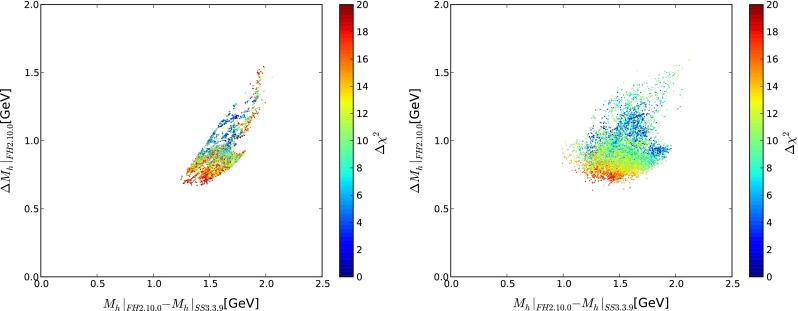
Scatter plots of 10000 points each selected randomly from scans [54] in the CMSSM (*left*) and the NUHM1 (*right*), displayed in $(M_h|_{\mathrm{FH}2.10.0} - M_h|_{\mathrm{SS}3.3.9}, \Delta M_h|_{\mathrm{FH}2.10.0})$ planes and colour-coded according to their $\chi ^2$ values

In the following we will compare FeynHiggs 2.10.0 with SoftSusy 3.3.9. While the higher-order corrections included in FeynHiggs 2.10.0 are more complete than those in SoftSusy, a very large discrepancy between the two codes would indicate a parameter region that is potentially unstable under higher-order corrections in at least one of the codes. Figure 7 displays planes of $M_h|_{\mathrm{FH}2.10.0} - M_h|_{\mathrm{SS}3.3.9}$ vs. the theoretical uncertainty $\Delta M_h|_{\mathrm{FH}2.10.0}$ estimated within FeynHiggs 2.10.0 (see [53] for details), displaying 10000 points chosen randomly from the samples in [54] (but with an upper limit on $\Delta \chi ^2 < 20$ to concentrate on the parts of parameter space of most phenomenological relevance) for the CMSSM (left panel) and the NUHM1 (right panel). The points are colour-coded according to the differences found in [54] between their $\chi ^2$ values and those of the best-fit points in the CMSSM and NUHM1, respectively, with low-$\Delta \chi ^2$ points in blue and high-$\Delta \chi ^2$ points in red.

The differences between the two codes are found in the region of $|M_h|_{\mathrm{FH}2.10.0} - M_h|_{\mathrm{SS}3.3.9}| = 1.0$–$2.0 \,\hbox {GeV}$ with a theoretical uncertainty prediction (for only the FeynHiggs calculation) between ${\sim }0.6$ and ${\sim }1.5$. The consistent difference between the two codes can be attributed to the more complete inclusion of higher-order corrections in FeynHiggs, which is reflected in the fact that the difference often exceeds the FeynHiggs theory uncertainty. On the other hand, no phenomenologically relevant parameter points are found with an unexpectedly large difference between the two codes. This indicates that the relevant parameter regions are not located in parts of the CMSSM/NUHM1 parameter space that lead to an unstable $M_h$ evaluation. This supports the viability of the constraints imposed by $M_h$ on these models.

**Fig. 8:**
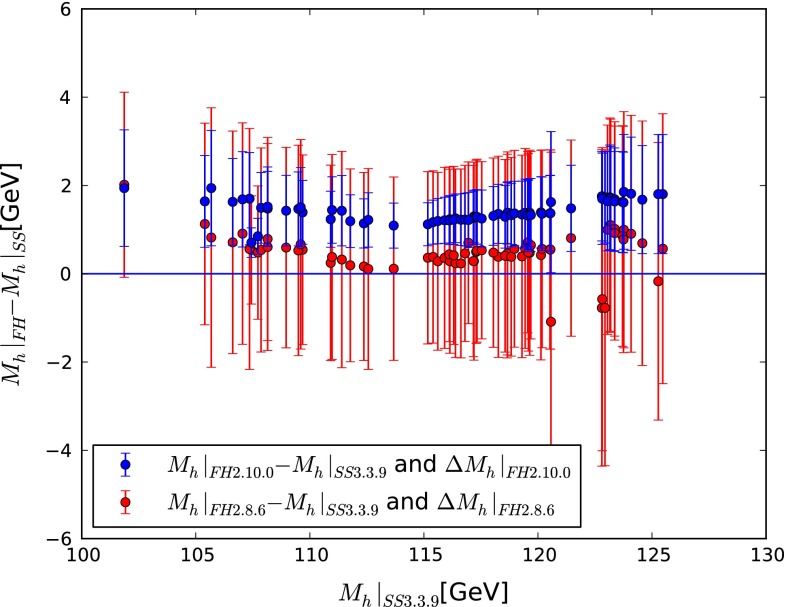
Values of $M_h|_{\mathrm{FH}2.10.0} - M_h|_{\mathrm{SS}3.3.9}$ (in *dark blue*) and of $M_h|_{\mathrm{FH}2.8.6} - M_h|_{\mathrm{SS}3.3.9}$ (in *red*) plotted against $M_h|_{\mathrm{SS}3.3.9}$, for 100 CMSSM points from the sample in [54] that have the lowest $\chi ^2$ for each bin in $M_h$. The *vertical lines* indicate the respective $M_h$ uncertainty calculations as evaluated by FeynHiggs

A similar inference can be drawn from Fig. 8. For this plot we have selected 100 CMSSM points from the sample in [54] that have the lowest $\chi ^2$ for each bin in $M_h|_{\mathrm{SS}3.3.9}$. We show their values of $M_h|_{\mathrm{FH}2.10.0} - M_h|_{\mathrm{SS}3.3.9}$ (in dark blue) and of $M_h|_{\mathrm{FH}2.8.6} - M_h|_{\mathrm{SS}3.3.9}$ (in red) on the vertical axis, using $M_h|_{\mathrm{SS}3.3.9}$ as the horizontal axis. In both cases the respective $M_h$ uncertainty calculations of FeynHiggs are indicated via vertical lines. We see that both FeynHiggs 2.10.0 and 2.8.6 yield values of $M_h$ that are systematically larger than SoftSusy 3.3.9. In most cases, $1 \,\hbox {GeV}\lesssim M_h|_{\mathrm{FH}2.10.0} - M_h|_{\mathrm{SS}3.3.9} \lesssim 2 \,\hbox {GeV}$, $0 \lesssim M_h|_{\mathrm{FH}2.8.6} - M_h|_{\mathrm{SS}3.3.9} \lesssim 1 \,\hbox {GeV}$ and $M_h|_{\mathrm{FH}2.10.0} - M_h|_{\mathrm{FH}2.8.6} \sim 1 \,\hbox {GeV}$. The change from version 2.8.6 to version 2.10.0 reflects the size of the newly included resummed corrections to $M_h$ for a relevant part of the parameter space.

The theoretical $M_h$ uncertainty evaluated in FeynHiggs 2.8.6 embraced the SoftSusy predictions as well as the updated FeynHiggs 2.10.0 prediction for $M_h$. The latter, in particular, gives confidence that the uncertainty calculation indeed captures the missing higher-order corrections. The new theoretical uncertainty as evaluated using FeynHiggs 2.10.0 does not include, in general, the older FeynHiggs prediction, nor does it include (in all cases) the SoftSusy prediction. This again demonstrates the effects and the relevance of the newly included resummed logarithmic corrections in FeynHiggs.

## Summary and conclusions

As we have shown in this paper, the improved Higgs mass calculations provided in the improved FeynHiggs 2.10.0 code have significant implications for the allowed parameter spaces of supersymmetric models. We have illustrated this point with examples in the pMSSM, CMSSM, NUHM1 and NUHM2 frameworks.

In a random scan of the pMSSM10 parameter space we exhibited the change in the Higgs mass $\Delta M_h$ in FeynHiggs 2.10.0 compared to the previous version FeynHiggs 2.8.6. This averages below 2 GeV for third family squark masses below 2 TeV, but it can increase up to $\Delta M_h\sim 5 \,\hbox {GeV}$ for $m_{\widetilde{q}_3}= 5 \,\hbox {TeV}$. The update to FeynHiggs 2.10.0 is therefore particularly relevant in light of the measured value of $M_h$ and the strengthened LHC lower limits on sparticle masses.

The CMSSM is under strong pressure from the LHC searches for jets + $/\!\!\!E_T$events, which exclude small values of $m_{1/2}$, the measurement of BR($B_s \rightarrow \mu ^+ \mu ^-$), which disfavours large values of ${\tan \beta }$, the measurement of $M_h$, which favours large values of $m_{1/2}$ and/or ${\tan \beta }$, and positive values of $A_0$, and the cosmological dark matter density constraint. We have shown that these constraints can be reconciled for suitable intermediate values of ${\tan \beta }$ if FeynHiggs 2.10.0 is used to calculate $M_h$ in terms of the input CMSSM parameters (with the exception of $(g-2)_\mu$). The pressure on the CMSSM would have been much greater if an earlier version of FeynHiggs had been used, which yielded lower values of $M_h$ because it did not include the leading and next-to-leading logarithms of type $\log (m_{\tilde{t}}/m_{t})$ in all orders of perturbation theory as incorporated in FeynHiggs 2.10.0.

The LHC constraints are satisfied more easily in the NUHM1 (and NUHM2), with their one (or two) extra parameters that offer more options for satisfying the cosmological dark matter density constraint at larger values of $m_{1/2}$ than in the CMSSM. The extra degree(s) of freedom in the NUHM1 (NUHM2) allow the Higgs mixing parameter $\mu$ or (and) $M_A$ to be adjusted so that a sizable Higgsino component is present increasing the annihilation cross section, and/or allowing $\chi \chi ^\pm$ and/or rapid direct-channel $\tilde{\chi }^0_{1}\tilde{\chi }^0_{1} \rightarrow H/A$ annihilation to bring the cosmological dark matter density into the allowed range. Reconciling all the constraints would have been possible already with the earlier version of FeynHiggs, but it is easier to achieve when the improved FeynHiggs 2.10.0 version is used.

In addition to the higher values of $M_h$ yielded by FeynHiggs 2.10.0, this code also provides a correspondingly reduced estimate of the theoretical uncertainty in the mass calculation. This must also be taken into account when analysing the consistency with other constraints within the CMSSM, NUHM1, NUHM2 or any other models. Taken together, the improved mass calculations and uncertainty estimates in FeynHiggs 2.10.0 make it a preferred tool for the analysis of supersymmetric models.

## Footnotes

1 Since the differences between the on-shell and $\overline{\mathrm{DR}}$ renormalisation in the scalar quark sector are formally of higher order, comparisons can be used to assess the uncertainties in the predictions of the Higgs mass.

2 We use here $\mathrm{BR}(b \rightarrow s \gamma )^\mathrm{exp} = (3.55 \pm 0.24) \times 10^{-4}$ i [62–67] in addition to a combined systematic and theory error of $0.13 \times 10^{-4}$. The green shaded region is excluded at 95 % CL. As established in previous studies of the CMSSM and NUHM, this constraint is typically more important for larger ${\tan \beta }$, negative $\mu$ and smaller $M_A$ and $(m_{1/2}, m_0)$.

3 These lines are drawn for a $(g-2)_\mu$ discrepancy of $(30.2 \pm 8.8) \times 10^{-10}$ [34, 68], corresponding to a combined $e^+ e^-$ estimate of the lowest-order hadronic polarisation contribution to the SM calculation of $(g-2)_\mu$. The $\tau$ decay data used to indicate a reduction in the discrepancy by about one $\sigma$ so that, for example, the outer solid lines in the figures would correspond approximately to the $- 1\sigma$ contours. However, a recent re-evaluation yields $\tau$ data results very similar to the $e^+e^-$ results [69].

4 The ATLAS $/\!\!\!E_T$ limit was quoted for the CMSSM with the choices ${\tan \beta }= 30$ and $A_0/m_0 = 2$, but a previous study [54] showed that such a contour is essentially independent of both ${\tan \beta }$ and $A_0/m_0$, as well as the amount of non-universality in NUHM models.

5 We take this opportunity to comment on the implications for fine-tuning of FeynHiggs 2.10.0. Since an LHC-compatible value of $M_h$ can be obtained for smaller values of $(m_{1/2}, m_0)$, other things being equal, the fine-tuning measure proposed in [71, 72] is generally reduced. For example, a point in the right panel of Fig. 3 that lies on the coannihilation strip and has a nominal value of $M_h= 125 \,\hbox {GeV}$ would require fine-tuning of ${\sim }700$ if FeynHiggs 2.10.0 is used, compared to ${\sim }1540$ if FeynHiggs 2.8.6 is used.

6 Here and in the lower left panel, in the black shaded region the LSP is a charged slepton other than the lighter stau.
